# RNA-Seq Study of Microbially Induced Hemocyte Transcripts from Larval *Heliothis virescens* (Lepidoptera: Noctuidae)

**DOI:** 10.3390/insects3030743

**Published:** 2012-08-14

**Authors:** Kent S. Shelby, Holly J. R. Popham

**Affiliations:** Biological Control of Insects Research Laboratory, USDA Agricultural Research Service, 1503 S. Providence Road, Columbia, MO 65203, USA; E-Mail: Holly.Popham@ars.usda.gov

**Keywords:** *Heliothis virescens*, transcription, RNA-seq, Illumina, innate immunity

## Abstract

Larvae of the tobacco budworm are major polyphagous pests throughout the Americas. Development of effective microbial biopesticides for this and related noctuid pests has been stymied by the natural resistance mediated innate immune response. Hemocytes play an early and central role in activating and coordinating immune responses to entomopathogens. To approach this problem we completed RNA-seq expression profiling of hemocytes collected from larvae following an *in vivo* challenge with bacterial and fungal cell wall components to elicit an immune response. A *de novo* exome assembly was constructed by combination of sequence tags from all treatments. Sequence tags from each treatment were aligned separately with the assembly to measure expression. The resulting table of differential expression had >22,000 assemblies each with a distinct combination of annotation and expression. Within these assemblies >1,400 were upregulated and >1,500 downregulated by immune activation with bacteria or fungi. Orthologs to innate immune components of other insects were identified including pattern recognition, signal transduction pathways, antimicrobial peptides and enzymes, melanization and coagulation. Additionally orthologs of components regulating hemocytic functions such as autophagy, apoptosis, phagocytosis and nodulation were identified. Associated cellular oxidative defenses and detoxification responses were identified providing a comprehensive snapshot of the early response to elicitation.

## 1. Introduction

Larvae of noctuid moths are major polyphagous pests of commodity crops such as maize, cotton, soybeans, alfalfa and vegetables throughout the world [1]. In North America and in Brazil larvae of the tobacco budworm, *Heliothis virescens* (F.) are major pests of agricultural production, feeding on cotton, maize, and soybean, as well as fruits, vegetables and ornamentals [2,3]. Control of *H. virescens *and other closely related heliothines would be advanced by understanding, and disrupting, the basic mechanisms underlying resistance to biological control agents such as entomopathogens and parasitoids, and identification of gene silencing targets for field applications [4,5,6]. However the molecular resources allowing fundamental laboratory and field research with the budworm to date are insufficient for this end [7,8,9]. 

Insects command an exquisitely evolved and powerful innate, *i.e*., germ-line encoded, immune response to microbial invasion which is divided between cellular and noncellular defenses [10]. Noncellular responses primarily involve the secretion of antimicrobial peptides, enzymes and other compounds into the hemocoel from immune-responsive tissues, while cellular responses are performed by several classes of hemocytes circulating within the hemocoel [11,12]. In order to effectively limit infection by microbes the innate immune system must first recognize microbial invasion, then accurately gauge the threat level presented by scattered microbial fragments, dead/nonviable, live but nonpathogenic, or live and pathogenic microbes (accurately distinguishing between invasive, commensal, mutualistic, symbiotic and pathogenic microbes); and finally, to mobilize and coordinate interacting humoral and cellular defensive components [13,14]. As classically described, an immune response is initiated against infectious non-self when Microbe-Associated Molecular Patterns (MAMPs; e.g., microbial peptidoglycans, lipopolysacharides, β–glucans, lipoproteins, CpG dinucleotides, or flagellins) bind to intracellular, transmembrane or extracellular Pattern Recognition Receptors (PRRs; e.g., *toll *receptors, C-type lectins, PGRPs, NOD-like or RIG-1-like receptors) in the presence of Damage-Associated Molecular Patterns (e.g., host nucleic acid, hyaluronan fragments, heat shock proteins, uric acid, ATP, or collagen fragments) [13,15]. Inducible antimicrobial peptides, inhibitors and enzymes are synthesized and released into the hemocoel [15,16]. Hemocytes become activated via PRR-linked signal transduction pathways then initiate degranulation, phagocytosis, microaggregation, nodulation, or encapsulation reactions [11,17]. Hemokine-, eicosanoid- and monoamine-mediated signaling pathways coordinate the behavioral, humoral and cellular innate responses against invading microbes [18,19,20].

Successful biological control of these pests with microbial entomopathogens requires measures that overcome or circumvent natural immunity. To better understand immune response against microbial entomopathogens we initiated a project to document the functional immunogenomics of *H. virescens* by identification of antimicrobial response orthologs to baculoviral, bacterial and fungal infection [8,21,22,23]. In this study we applied the technique of Illumina RNA-seq digital expression profiling to construct a hemocyte-specific *de novo* transcriptome, comprising >22,000 putative transcripts, against which we profiled the *in vivo* antimicrobial responses of hemocytes infected with bacterial or fungal elicitors within the first hours of infection. 

## 2. Results and Discussion

### 2.1. Transcriptome Assembly

In this report we describe RNA-seq expression profiling of larval *H. virescens* hemocyte response to microbial elicitation *in vivo*. Hemocytes were collected from larvae following elicitation with bacterial or fungal cell wall components and the resulting hemocyte transcripts were sampled by collecting three technical replicate lanes of 42-base sequence tags from each treatment using the Illumina GAIIx platform yielding a total 6,239,031 kb of reads filtered of ribosomal and mitochondrial sequences. A hemocyte-specific *de novo* transcriptome assembly was constructed by combining these 42-base tags from all treatments and controls into a single assembly using VELVET/OASIS resulting in 22,007 contigs, each with a distinct combination of annotation and expression (mean length, 765 bp; median length, 391 bp; range, 101 to 18,452 bp). When assemblies without useful annotations were excluded 13,216 assemblies remained. The 81,872 assemblies without a significant BLAST score or annotation were excluded from the present analysis. Within this larval *H. virescens* hemocyte *de novo* transcriptome assembly we identified *H. virescens* orthologs of known metazoan genes by BLASTx against the NCBI NR database (cutoff e < 10^−6^). Of the 35,959 top BLAST hits 30,249 were arthropod species; 30,020 were insecta, 29,205 endopterygotes; 7,135 dipterans; 6,539 coleopterans; and finally 6,314 were specific to lepidopteran species [24]. 

The larval *H. virescens de novo* exome assembly contained a full complement of aminoacyl-tRNA-synthetase transcripts along with a partial complement of mitochondrial aminoacyl-tRNA synthetases. Within the 311 assemblies identified by genome ontology as mitochondrial were the majority of enzymes involved in oxidative phosphorylation. Additional enzymes of central metabolism were identified including those involved in amino acid, lipid and carbohydrate biosynthesis and transport, vitamin and cofactors synthesis, xenobiotic biodegradation, and purine metabolism. Transcripts annotating within the ubiquitin-proteasome cellular degradation pathway (346), mitochondrial metabolism, and protein synthesis were identified which comprised the majority of components within each category. Cytoskeletal components such as tubulins (70), actins (49), myosins, spectrins, and microtubules (38) were noted. Of particular interest for future studies, a comprehensive range of hemocytic plasma membrane proteins were identified including ion channels and transporters, G-protein coupled receptors (46), transcription factors, amino acid/carbohydrate transporters, aquaporins (6), ABC multidrug resistance transporters (26), ankyrins (27), annexins (11), cell surface receptors, 14-3-3 proteins (6), gap junctions (8) and components of the endo/exocytosis, peroxisomal and lysosomal compartments. Neuropeptides, surface and nuclear neurotransmitter and hormone receptors and enzymes involved in endocrine regulation via ecdysteroids (16) and juvenile hormones (25) were present. Retrotransposon sequences were identified within the assembly (156), as were assemblies homologous to proteins of the bracovirus and other viral families, phages, mycoplasma and other bacterial proteins. Possible low level contamination with other tissues may be indicated by the presence of transcripts for the storage proteins arylphorins, riboflavin binding hexamer, methionine rich storage protein, vitellogenin, lipophorin and putative cuticular proteins. Alternatively small amounts of these proteins may also be transcribed in *H. virescens* hemocytes. 

**Figure 1:**
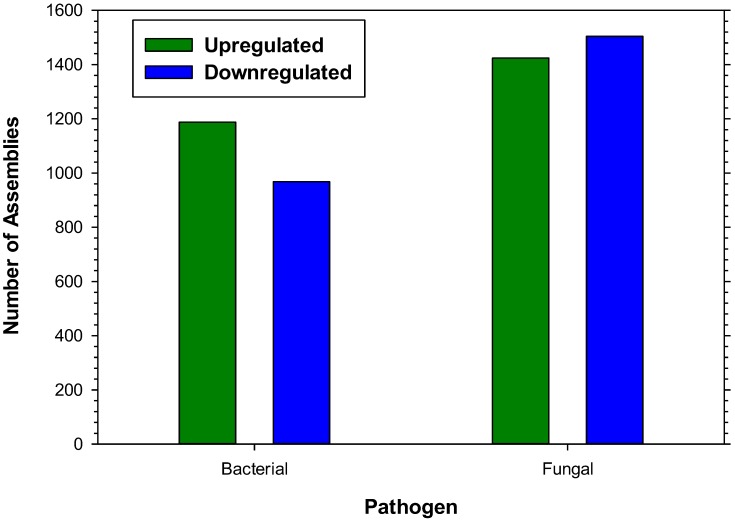
Number of *H. virescens* larval hemocyte transcripts upregulated (**green**) or downregulated (**blue**) more than 3-fold by elicitation with either bacterial or fungal cell wall components.

Expression profiling of microbial responses was accomplished by aligning the 42-base sequence tags from each treatment to the *de novo* reference assembly, comparing each treatment to control levels of expression. Elicitation of the immune response of *H. virescens* larvae with a mixture of Gram^+^/Gram^−^ bacterial cell wall components resulted in at least a 3-fold upregulation of 1,188 hemocyte assemblies and 3-fold downregulation of 968 (Figure 1). Elicitation with fungal cell wall components resulted in the upregulation of 1,424 and the downregulation of 1,504 hemocyte assemblies (Figure 1). More than 700 assemblies corresponding to known orthologs of immune system were identified and those exhibiting 3-fold alterations in expression level following microbial challenge are presented (Table 1, Table 2, Table 3 and Table 4). Fungal treatment resulted in more than 3-fold upregulation of 1,191 assemblies and downregulation of 1,425 when compared to the bacterial treatment. Conversely, bacterial elicitation resulted in greater than 3-fold upregulation of 1,482 and downregulation of 1,134 assemblies when compared to the fungal elicitation. Annotated assemblies showing alteration of transcriptional expression levels following immune elicitation are discussed in separate categories below.

**Table 1:** Pattern Recognition and Signal Transduction.

Gene ID		Contig ID(HvUMC.)	Contr	Bac	Bac Fold Change	Fung	Fung Fold Change	Contig Length	% Cov	SpeciesTop Blast	e-Value
**Up-Regulated Genes**											
	β-1,3-Glucan Recognition Protein 2b	2.6025.Contig1	47	138	2.9	146	3.1	239	77	*Helicoverpa armigera*	4E-27
	C-type Lectin 10	2.28786.Contig1	32	112	3.5	92	2.9	487	52	*Bombyx mori*	2E-38
	C-type Lectin	1.single.v21-20824	1,723	4,150	2.4	7,281	4.2	3,126	46	*Aedes aegypti*	E-122
	Cyclophilin	1.single.v31-289707	4	19	4.8	13	3.3	591	53	*Aedes aegypti*	1E-50
	Dscam, Isoform AY	1.single.v21-784756	6	12	2.0	18	3.0	159	84	*Drosophila melanogaster*	4E-17
	Dscam	1.single.v21-781454	6	14	2.3	26	4.3	192	92	*Pediculus h. corporus*	5E-27
	F-box/LRR-repeat protein 16	1.single.v21-728991	2	8	4.0	12	6.0	187	88	*Camponotus floridanus*	4E-24
	Galactose Binding Lectin, Soluble 9	1.single.Rem21-3740	142	508	3.6	194	1.4	696	51	*Mus musculus*	1E-19
	Hemocytin	1.single.Rem27-385	1,152	3,816	3.3	5,276	4.6	484	39	*Harpegnathos saltator*	8E-31
	Immulectin-2	6.26705.Contig1	66	58	0.9	844	12.8	1,005	36	*Manduca sexta*	1E-49
	Immulectin-3	1.single.v23-434605	17	51	3.0	44	2.6	235	60	*Manduca sexta*	1E-6
	Integrin alpha 3	3.22533.Contig1	1,133	2,519	2.2	4,186	3.7	3,312	60	*Pseudoplusia includens*	0
	Integrin Beta 1 Subunit	1.single.Rem23-17883	31	126	4.1	66	2.1	130	100	*Spodoptera exigua*	3E-12
	Integrin-linked Kinase	4.1761.Contig1	31	96	3.1	102	3.3	633	78	*Glossina morsitans*	1E-63
	Lectin 3	1.single.v21-686389	11	43	3.9	27	2.5	245	36	*Lonomia obliqua*	2E-8
	Leucine Rich Protein	1.single.v37-139619	8	15	1.9	32	4.0	227	36	*Aedes aegypti*	2E-8
	Lipopolysaccharide Binding Protein	3.9650.Contig1	2,442	7,490	3.1	8,921	3.7	158	78	*Helicoverpa armigera*	1E-11
	NF-Kappa B Essential Modulator	1.single.Rem21-38499	12	39	3.3	24	2.0	176	48	*Aedes aegypti*	4E-6
	Paralytic Peptide Binding Protein 2	64.547.Contig4	3	197	65.7	1	0.33	247	42	*Bombyx mori*	7E-5
	Peroxinectin	2.12813.Contig1	6	25	4.2	32	5.3	190	45	*Ixodes scapularis*	2E-7
	PGRP	6.1191.Contig1	302	1,969	6.5	6,633	22.0	1,366	87	*Helicoverpa armigera*	3E-88
	PGRP-SA	1.single.v37-159335	71	171	2.4	249	3.5	232	78	*Tribolium castaneum*	3E-13
	Scavenger Receptor	1.single.v29-351108	3	9	3.0	14	4.7	172	71	*Culex quinquefasciatus*	2E-18
	Toll Precursor	15.16134.Contig1	178	550	3.1	708	4.0	2,095	28	*Pediculus h. corporis*	4E-58
	Toll receptor 18-Wheeler	1.single.v37-162137	32	41	1.3	98	3.1	144	52	*Spodoptera frugiperda*	7E-5
**Down-Regulated Genes**											
	Galectin-12	9.8877.v39-13988	139	358	2.6	11	0.08	218	48	*Harpegnathos saltator*	3E-12
	Lectin 5	17.2309.Contig1	266	36	0.14	361	1.4	848	45	*Lonomia obliqua*	2E-36
	NADP-Leukotriene B4 12- hydroxydehydrogenase	1.single.v25-664428	12	5	0.42	2	0.17	424	63	*Culex quinquefasciatus*	2E-45

Bac, Bacterial infected samples; Fung, Fungal infected samples; % Cov, percent query coverage; e-value, expect value.

**Table 2:** Melanization and Coagulation.

Gene ID		Contig ID(HvUMC.)	Contr	Bac	Bac Fold Change	Fung	Fung Fold Change	Contig Length	% Cov	SpeciesTop BLAST	e-Value
**Up-Regulated Genes**											
	Brasiliensin Thrombin Inhibitor	1.single.v23-492316	114	195	1.7	453	4.0	792	41	*Triatoma basiliensis*	6E-32
	DOPA Decarboxylase	1.single.v21-586620	76	120	1.6	621	8.2	1,394	94	*Mamestra brassicae *	0
	Hemocyte Protein-Glutamine Gamma- Glutamyltransferase	1.single.v23-275987	232	374	1.6	1,449	6.2	2,292	34	*Camponotus floridanus*	0
	Hemolymph Proteinase 18	3.3815.Contig1	490	710	1.4	4,941	10.1	1,350	41	*Manduca sexta*	8E-74
	Hemolymph Proteinase 19	1.single.v21-731703	14	54	3.9	22	1.6	369	69	*Manduca sexta*	2E-49
	Immulectin-2	6.26705.Contig1	66	58	0.9	844	12.8	1,005	36	*Manduca sexta*	1E-49
	Immulectin-3	1.single.v23-434605	17	51	3.0	44	2.6	235	60	*Manduca sexta*	1E-6
	Laccase-4-like	1.single.Rem21-26133	6	31	5.2	13	2.2	292	56	*Bombus terrestris*	1E-22
	Laccase-7-like	2.7276.Contig1	9	33	3.7	14	1.6	269	55	*Acyrthosiphon pisum*	8E-23
	Prophenoloxidase Activating Enzyme	3.18472.Contig1	40	166	4.2	168	4.2	135	95	*Helicoverpa armigera*	4E-16
	Reeler	1.single.v21-527354	7	189	27.0	549	78.4	502	77	*Bombyx mori*	1E-61
	Serpin-like Protein	1.single.v21-800532	2	4	2.0	20	10.0	167	83	*Antheraea mylitta*	7E-19
	Transglutaminase	1.single.Rem21-50794	2	6	3.0	13	6.5	185	76	*Apis mellifera*	2E-18
	Yellow 2	1.single.Rem21-90085	2	1	0.5	11	5.5	124	57	*Bombyx mori*	6E-6
	Yellow-b	1.single.v23-9110	832	2,954	3.6	2,802	3.4	1,833	78	*Heliconius melpomene*	0
	Yellow-f	1.single.v39-31578	49	147	3.0	154	3.1	559	85	*Bombyx mori*	6E-92
**Down-Regulated Genes**											
	Hemolymph Proteinase 17	1.single.v21-676209	395	19	0.05	72	0.18	1,979	51	*Manduca sexta*	1E-168
	Hemolymph Proteinase 20	2.20519.Contig1	15	3	0.20	4	0.27	253	51	*Manduca sexta*	3E-17
	Phenylalanine Hydroxylase	1.single.v25-669762	14	3	0.21	2	0.14	250	95	*Papilio xuthus*	3E-41

Bac, Bacterial infected samples; Fung, Fungal infected samples; % Cov, percent query coverage; e-value, expect value.

**Table 3:** Antimicrobials And Effectors.

Gene ID		Contig ID (HvUMC.)	Contr	Bac	Bac Fold Change	Fung	Fung Fold Change	Contig Length	% Cov	Species Top BLAST	e-Value
**Up-Regulated Genes**											
	Antibacterial Protein 3Tox	35.7580.Contig4	63	1,115	17.7	2,810	44.6	270	77	*Heliothis virescens*	1E-24
	Antimicrobial Protein 5Tox	24.13350.Contig4	16	451	28.2	538	33.6	314	64	*Bombyx mori*	2E-27
	Antimicrobial Protein 6Tox	24.13350.Contig5	16	273	17.1	766	47.9	267	55	*Bombyx mori*	2E-19
	Attacin A Precursor	3.25069.Contig1	4	156	39.0	587	146.8	735	100	*Heliothis virescens*	E-68
	Carboxylesterase	3.2554.Contig1	9	49	5.4	92	10.2	445	87	*Helicoverpa armigera*	3E-70
	Cecropin 3	34.474.Contig1	123	1,336	10.9	1,333	10.8	366	86	*Helicoverpa armigera*	3E-31
	Cecropin A2	34.474.Contig3	275	917	3.3	598	2.2	301	66	*Helicoverpa armigera*	3E-14
	Cecropin D	11.9800.Contig1	25	246	9.8	411	16.4	345	74	*Helicoverpa armigera*	2E-17
	Chymotrypsin Inhibitor CI-8A	1.single.v37-179791	285	450	1.6	1,453	5.1	493	71	*Bombyx mori*	3E-65
	Cobatoxin B Long Form	1.single.v29-23061	2,365	25,592	10.8	21,264	9	555	51	*Spodoptera frugiperda*	9E-16
	Gallerimycin	6.18346.Contig1	21	372	17.7	846	40.3	362	78	*Helicoverpa armigera*	9E-27
	Gloverin-like Antibacterial Protein	5.6087.Contig1	13	420	32.3	645	49.6	426	82	*Heliothis virescens*	5E-68
	Hemolin	3.4762.Contig1	313	3,201	10.2	6,907	22.1	1,239	96	*Heliothis virescens*	0
	Immune Inducible Protein	3.5943.Contig1	38	759	20.0	1,773	46.7	294	79	*Helicoverpa armigera*	2E-22
	Inducible Metalloproteinase Inhibitor	1.single.v21-505063	133	518	3.9	178	1.3	693	53	*Galleria mellonela*	1E-17
	I-type Lysozyme	3.8206.Contig1	290	509	1.8	1,038	3.6	795	57	*Sitophilus zeamais*	6E-42
	Heliocin Precursor	12.13910.Contig1	26	361	13.9	813	31.3	583	86	*Heliothis virescens*	3E-49
	Kazal-type Inhibitor	2.5500.Contig1	4	21	5.3	5	1.3	351	48	*Panstrongylus megistus*	6E-20
	Lysozyme	3.27236.Contig1	6,536	16,912	2.6	23,565	3.6	355	99	*Heliothis virescens*	3E-67
	Metalloproteinase Inhibitor 3	1.single.v21-774328	56	102	1.8	211	3.8	1,054	42	*Tribolium castaneum*	2E-39
	Nimrod-like Protein	4.7692.Contig1	315	1,043	3.3	2,122	6.7	843	47	*Tribolium castaneum*	2E-47
	Viresin	2.22031.Contig1	28	35	1.2	194	6.9	448	90	*Heliothis virescens*	9E-61
	Virescein Precursor	2.19097.Contig1	0	19	19.0	65	65.0	193	100	*Heliothis virescens*	3E-6
**Down-Regulated Genes**											
	Adamts-7 Metallopeptidase	1.single.v37-182137	33	6	0.18	3	0.09	167	69	*Aedes aegypti*	7E-16
	Chemosensory Protein	1.single.Rem23-43567	22	3	0.14	19	0.87	304	100	*Heliothis virescens*	4E-33
	Immune-related Hdd13	3.8673.Contig1	106	28	0.26	18	0.17	340	56	*Hyphantria cunea*	1E-31
	Odorant Binding Protein	1.single.v29-513279	26	2	0.08	30	1.15	179	98	*Heliothis virescens*	3E-27

Bac, Bacterial infected samples; Fung, Fungal infected samples; % Cov, percent query coverage; e-value, expect value.

**Table 4:** Cellular Stress Response.

Gene ID		Contig ID(HvUMC.)	Contr	Bac	Bac Fold Change	Fung	Fung Fold Change	Contig Length	% Cov	SpeciesTop BLAST	e-Value
**Up-Regulated Genes**											
	Antennal Cytochrome P450 CYP4	1.single.v37-177294	5	49	9.8	1	0.20	271	78	*Mamestra brassicae*	2E-28
	Antennal Cytochrome P450 CYP9	1.single.v27-352481	27	32	1.2	280	10.4	701	78	*Mamestra brassicae*	1E-103
	Autophagy-like Protein Atg12	2.26567.Contig1	4	9	2.3	17	4.3	265	69	*Biston betularia*	1E-10
	Autophagy-related Protein B2-like	1.single.v29-386662	2	6	3.0	8	4.0	714	50	*Acromyrmex echinatior*	6E-51
	Autophagy-related Protein 9A	2.9906.Contig1	3	8	2.7	15	5.0	170	60	*Danio rerio*	3E-13
	Calcium & Integrin-binding Protein 1	1.single.v35-260454	11	33	3.0	19	1.7	231	61	*Harpegnathos saltator*	1E-13
	Calnexin 99A, Isoform C	1.single.v39-34103	29	96	3.3	81	2.8	241	73	*Drosophila melanogaster*	1E-30
	Calpain Protein	6.18445.Rem27-3414	42	126	3.0	90	2.1	205	85	*Bombyx mori*	1E-5
	Calponin/Transgelin	3.3375.Contig1	121	134	1.1	466	3.9	854	91	*Aedes aegypti*	9E-84
	CarE	1.single.v21-683558	167	271	1.6	536	3.2	152	80	*Spodoptera exigua*	0
	Carboxyl/choline Esterase	1.single.v25-663787	11	48	4.4	58	5.3	242	75	*Helicoverpa armigera*	6E-30
	Catalase	1.single.Rem23-39568	39	58	1.5	384	9.8	152	92	*Spodoptera litura*	3E-15
	Fasciclin-1	1.single.v29-874	3489	29527	8.5	15189	4.4	1459	56	*Danaus plexippus*	5E-94
	Ferritin Heavy Chain	1.single.v23-133164	10	60	6.0	0	0	159	92	*Trichoplusia ni*	8E-20
	Glutathione Synthetase	3.11637.Contig1	5	36	7.2	16	3.2	275	60	*Harpegnathos saltator*	1E-6
	Glutathione S-transferase	1.single.v21-548409	16	72	4.5	238	14.9	639	73	*Amyelois transitella*	2E-86
	Gossypol-induced Cytochrome P450	1.single.v35-24142	3111	13869	4.5	5967	1.9	1908	79	*Helicoverpa armigera*	0
	Hemicentin-like Protein 2	1.single.v33-251	235	1084	4.6	651	2.8	363	50	*Spodoptera frugiperda*	2E-20
	Innexin 1	1.single.Rem21-52588	76	243	3.2	359	4.7	200	82	*Pediculus h. corporis*	1E-25
	Innexin 3	3.4996.Rem23-8776	35	168	4.8	245	7.0	263	68	*Harpegnathos saltator*	5E-25
	Laminin A Chain	2.31786.Contig1	100	317	3.2	142	1.4	844	69	*Aedes aegypti*	2E-64
	Laminin-like Protein Epi-1	1.single.v25-592818	11	34	3.1	40	3.6	236	58	*Camponotus floridanus*	2E-16
	NADPH Oxidase 5-like	1.single.v21-32103	31	143	4.6	187	6.0	382	77	*Acyrthosiphon pisum*	2E-49
	Papilin	3.16799.Contig1	120	742	6.2	556	4.6	1366	40	*Harpegnathos saltator*	5E-63
	Peroxidasin	1.single.v21-283343	806	1896	2.4	2986	3.7	4327	50	*Tribolium castaneum*	0
	Selenium-binding protein	2.31409.Contig1	154	388	2.5	785	5.1	355	59	*Pediculus h. corporus*	8E-30
	Talin-2-like	2.27492.Contig1	26	63	2.4	89	3.4	1280	75	*Bombus terrestris*	1E-178
	Teneurin-3 Isoform 1	1.single.v21-374519	217	652	3.0	703	3.2	597	88	*Apis mellifera*	4E-98
	Transferrin	4.13853.Contig1	28	101	3.6	187	6.7	1071	33	*Aedes aegypti*	2E-44
**Down-Regulated Genes**											
	Apoptosis Inducing Factor, Putative	1.single.v21-402220	272	286	1.1	54	0.20	391	62	*Ixodes scapularis*	7E-16
	Calnexin, Putative	1.single.Rem21-38232	26	18	0.69	5	0.19	159	68	*Ixodes scapularis*	1E-14
	Ferrochelatase precursor	1705.v27-585704	16	3	0.188	3	0.188	149	66	*Chironomus sp.*	7E-11
	Flavin-dependent Monooxygenase	1.single.v21-786790	79	24	0.30	36	0.46	501	97	*Helicoverpa armigera*	2E-87

Bac, Bacterial infected samples; Fung, Fungal infected samples; % Cov, percent query coverage; e-value, expect value.

### 2.2. Pattern Recognition Receptors

PRRs act as sentinels to microbial incursion by first binding to MAMPs, then activating specific signal transduction pathways. A number of *H. virescens* orthologs of different PRR classes were identified among the hemocyte transcripts. Hemocyte assemblies orthologous to the β-1,3-glucan recognition protein family which includes Gram^−^ bacteria-binding protein were identified (Table 1). Peptidoglycan binding recognition protein (PGRP) transcript levels were upregulated by microbial elicitation (Table 1). Small secreted PGRP-Small secreted A (PGRP-SA) forms bind Gram^+^ bacteria, activating the *toll* pathway via cleavage of Spätzle; and Gram^-^ recognizing PGRP-long transmembrane F form activating the *imd* pathway were elevated following bacterial or fungal elicitation of larvae (Table 1). Representatives of the Ca^+2^-dependent C-type lectins were identified including lipopolysaccharide binding protein and orthologous assemblies of immulectins-2, and -3. In these expression profiling experiments C-type lectins were also induced by bacterial elicitation, as was previously observed using quantitative RT-PCR of *H. virescens* hemocytes [8]. The class of galactose binding lectins and galectins, was also represented among the annotated assemblies, as were hemocytin and additional lectins of various classes (Table 1).

Additional PRRs involved in recognition of microbial infection and upregulated by elicitation were attacin, hemolin, scolexin, and scavenger receptors (Table 1). Transcripts of the lipopolysaccharide-binding leucine rich repeat protein leureptin were identified. Leureptin associates with *M. sexta* hemocytes and interacts with MD-2-related lipid recognition protein to effect lipopolysaccharide activation of the *toll *signaling pathway [25]. Leucine rich repeat proteins were upregulated by either fungal or bacterial elicitation, or both (Table 1). Integrin is a cell surface pattern recognition receptor which can for example recognize collagen IV fragments associated with tissue damage [15]. *Per os* gene silencing of a *Plutella xylostella* β-integrin inhibited *E. coli* stimulated hemocyte nodulation and also caused excess larval mortality [26]. *H. virescens* hemocytic integrin transcription was upregulated by bacterial and fungal elicitation (Table 1). Expression of *H. virescens* hemocytic neuroglian and tetraspanin orthologs was not altered by microbial infection. 

Several assemblies orthologous to the Down syndrome cell adhesion molecule (Dscam) [27] PRR were identified among the *H. virescens* hemocyte transcripts (Table 1). Dscam are expressed from a single gene with multiple exons and are processed into thousands of variants via differential splicing and participate in phagocytosis and nervous system development. Surprisingly few Dscam orthologous transcripts were identified in *H. virescens* hemocytes when compared to *Drosophila melanogaster* which is known to generate more than 38,000 splice variants [27]. More detailed studies using this same dataset will become possible when a tiling array of the *H. virescens* Dscam gene is constructed. 

One final class of putative insect PRRs, the circulating plasma lipoprotein particle lipophorin and its associated protein apolipophorin-III, were identified within our larval hemocyte transcriptome but exhibited no alteration in expression levels. In *Bombyx mori* and other insects lipophorin and apolipophorin-III serve as PRRs for β–1,3-glucans, lipopolysaccharide, and lipoteichoic acid [28]. Additionally, lipophorin may be directly microbicidal [29] and apolipophorin-III exhibits antiplasmodial activity [30]. 

PRR activation at remote locations can be signaled to immune responsive tissues such as hemocytes *via* peptide chemokines [11]. Orthologs of proteins involved in this activation process were identified within the *H. virescens* transcripts including hemocyte aggregation inhibitor, macrophage migration inhibitory factor, imaginal disc growth factor, and paralytic peptide binding proteins-1 and -2. Growth blocking peptide circulating in the hemolymph is proteolytically processed to the active peptide following stressors such as heat, injury and bacteria in *Drosophila*, activating antimicrobial peptide synthesis via the Gram- sensitive *imd* pathways [31]. Expression of the identified *H. virescens* hemocytic assemblies orthologous to growth-blocking peptide and growth blocking peptide binding protein were unaffected by microbial elicitation, in agreement with our previous work [8]. Octopamine and 5-hydroxytryptamine have also been implicated as modulators of hemocyte activation [18], and we identified assemblies of *H. virescens* hemocyte neurotransmitter receptors orthologous to both which were not upregulated by immune elicitation. Larval *Chilo suppressalis* hemocyte octopamine transporters were also not upregulated by fungal or bacterial injections [18].

### 2.3. Signal Transduction

Insect immune-responsive tissues respond to microbial activation of PRRs via several signal transduction pathways, the most well studied of which are the Gram^+^ and fungal responsive *toll* pathway, the Gram^-^ responsive *imd*-JNK pathway, and the JAK/STAT pathway [16]. In this study of immune-stimulated *H. virescens* hemocytes over 1200 assemblies annotating as signal transduction pathway components were present (e.g., apoptosis, *toll*, *imd*, JAK/STAT, prostaglandin, phosphatidylinositol 3-kinase, insulin/IGF/mTOR, G-protein coupled receptors, adenylate/guanylate cyclases, calreticulin, *Rac*, *ras*, protein kinases/phosphatases, phospholipases, *etc.*) however only a very few of these assemblies exhibited alterations in their expression levels as a result of the treatments employed. Therefore, only those whose transcription was upregulated more than 3-fold by the treatments are shown in Table 1. 

Immune activation of lepidopteran hemocytes *via* eicosanoid intermediates has been well documented [20,32]. MAMP binding by PRRs in hemocytes or fat bodies may cause the release of plasmatocyte spreading peptide which in turn activates a hemocytic signal transduction pathway whereby the C20 fatty acid arachidonic acid is cleaved from the inner leaflet of cells by an activated phospholipase A_2_ (PLA_2_), and is subsequently oxidized to bioactive eicosanoids such as prostaglandins by cytosolic cyclooxygenase or lipoxygenase. Hemocyte actions mediated by the PLA_2_ signaling pathway include phagocytosis, cellular spreading, microaggregation and nodulation as well as prophenoloxidase activation [32]. Inhibition of lepidopteran PLA_2_ either by chemical means, gene silencing, or by entomopathogenic bacteria leads to profound immunosuppression [32]. Several *H. virescens* orthologs of enzymes within this transduction pathway were identified, among them a PLA_2_, a secretory PLA_2_, PLA_2_-activating protein, prostaglandin E synthase 2, prostaglandin reductase 1, prostamide/prostaglandin F synthase, NADP-dependent leukotriene B4 12-hydroxydehydrogenase, leukotriene A4 hydrolase, and peroxinectin. Of these orthologs only two, peroxinectin (upregulated 5×) and NADP-dependent leukotriene B4 12-hydroxydehydrogenase (downregulated 6×) exhibited differential regulation in hemocytes following bacterial or fungal elicitation (Table 1). Interruption of hemocytic signal transduction pathways mediated *via* eicosanoids using RNAi [32] may now be a feasible approach in *H. virescens* using the sequences discussed above. These newly identified orthologs may prove useful in teasing out the multiple interacting regulatory pathways coordinating the innate cellular immune responses of *H. virescens* hemocytes against entomopathogens.

### 2.4. Melanization, Nodulation and Encapsulation

The enzyme prophenoloxidase (PPO) is proteolytically activated by a cascade of serine protease initiated by PRR activation [12]. Expression of the *H. virescens* PPO-1, and PPO-2 assemblies were not altered by microbial elicitation. This confirms our earlier report [22] in which the expression of these key plasma multicopper oxidases was not affected by bacterial or baculoviral infection. The majority of components of the PPO activation pathway isolated from *Manduca sexta* were identified in this survey, including PPO activating enzymes, immunolectins-2 and -3, and hemolymph proteinases-6, and -21, Spätzle cleaving proteinase-8 (Table 2) [12]. Assemblies orthologous to *M. sexta *pattern recognition serine proteinase precursor (proHP14) were identified, however in our experiments no upregulation was observed upon bacterial or fungal elicitation. Serpins involved in throttling the above serine protease PPO activating cascade were also identified which were upregulated following fungal elicitation (Table 2), while pacifastin-related serine protease inhibitor precursor was not. Enzymes preceding PPO in the melanization pathway, phenylalanine hydroxylase, and following PPO in the pathway, DOPA decarboxylase, arylalkylamine N-acetyltransferase and laccases were present (Table 2). Hemolymph clotting cascade orthologs were present (Table 2). Upon bacterial elicitation *H. virescens* hemocytes release a β-amyloid-like protein (p102) which participates in melanization and encapsulation reactions [33]. Assemblies matching p102 were identified within the hemocyte transcriptome, along with amyloid binding proteins; however no change in their transcript levels was detected upon microbial elicitation. A reelin-domain assembly orthologous to the *B. mori* Reeler involved in nodulation reactions was highly upregulated by both bacterial and fungal elicitation [34] (Table 2). Orthologs to the EGF repeat containing phagocytosis regulating peptides draper and eater were identified within this assembly but expression levels were unaffected by the treatments. Formation of the invertebrate form of gap junctions between cells is accomplished by adjoining hexamers of innexins (invertebrate connexins) during nodulation and encapsulation reactions of *H. virescens* hemocytes [35]. Within our *de novo* transcriptome we identified *H. virescens* orthologs of innexins-1 and -3 both of which were upregulated by bacterial (4.8-fold) and fungal (7-fold) elicitation (Table 4).

### 2.5. Antimicrobials and Other Effectors

Activation of the *toll*, *imd* signal transduction pathways upregulates transcription of a suite of typically cationic antibacterial and antifungal antimicrobial peptides by hemocytes, fat bodies, Malpighian tubules, midgut, reproductive, cuticular and other tissues [16,36]. Antimicrobial peptides previously isolated from tissues of immune-stimulated *H. virescens* larvae include cecropins, heliocin [37], attacin, virescein, and heliomicin [38]. Cecropins are a class of amphipathic antibacterial peptides widespread in nature [16]. Three cecropins have been chromatographically purified from *H. virescens* plasma, and transcript assemblies corresponding to each were identified among the upregulated elements in this study (Table 3). Transcription of the peptide virescin, first isolated from immune-stimulated larval *H. virescens* plasma [39], matches an assembly which was upregulated 7-fold in hemocytes by fungal elicitation (Table 3). Transcription of the moricin-like antibacterial peptide virescein was highly upregulated in hemocytes by both bacterial (19-fold) and fungal (65-fold) treatments (Table 3). An assembly corresponding to heliocin, a proline-rich lebocin-like glycosylated *H. virescens* antibacterial peptide [38], was upregulated by both classes of microbial elicitations. Substantial upregulation of the glycine-rich antibacterial peptides gloverin and attacin occurred following microbial elicitation (Table 3). 

The defensin related peptide heliomicin exhibited only antifungal activity even though its synthesis was induced by septic injury with a suspension of gram positive and gram negative bacteria [38]. However the assembly corresponding to heliomicin precursor exhibited no upregulation by either bacterial or fungal treatment in our experiments. Instead a related defensin annotating as gallerimicin-like was highly upregulated by microbial elicitation (Table 3). Additional defensin-related antimicrobial peptides were identified within our assembly, including x-Tox peptides found only in Lepidoptera. These peptides encode variable repeats of a cysteine stabilized alpha-beta sheet (CS- αβ) scorpion toxin related motif [40]. An earlier EST effort identified Hv-3-Tox in transcripts from immune-stimulated larval *H. virescens* hemocytes which encoded three of these motifs [8]. The *S.frugiperda* ortholog of this protein, encoding 11 CS-αβ-motifs was expressed solely in the granulocytes and plasmatocytes [40,41]. In agreement with these authors we observed significant upregulation of these *H. virescens* X-tox assemblies in our hemocyte transcript survey (Table 3). The *H. virescens* hemocytic orthologs of the *Drosophila* 14-3-3ε necessary for the secretion of antimicrobial peptides from hemocytes [42] were identified, but did not exhibit differential regulation by either fungal or bacterial elicitation.

Infection induces the synthesis of host antimicrobial proteases and protease inhibitors targeted against microbial virulence factors [43]. In these experiments inducible metalloproteinase inhibitor proteins were upregulated in hemocytes by bacterial but not by fungal elicitation (Table 3). Orthologs of a pacifastin-like serine protease inhibitor along with several other uncharacterized serine and cysteine protease inhibitors were identified in this study. Two metalloprotease inhibitors were significantly upregulated by either bacterial or fungal elicitation (Table 3). The effectors attacin and hemolin were also significantly upregulated by microbial infection. Attacin transcription was elevated 39-fold by bacterial elicitation and 146.8-fold by fungal elicitation (Table 3). In this report hemolin upregulation observed 10.2-fold in bacterially elicited hemocytes and 22.1-fold in fungally elicited larval hemocytes. These values are somewhat greater than the range previously demonstrated using quantitative RT-PCR in bacterially elicited *H. virescens* larval hemocytes and fat bodies [21].

### 2.6. RISC Antiviral Complex

Hemocytes expressed transcripts corresponding to components of the antiviral RISC complex vital to innate immunity against RNA virus infection [44] were identified, including argonautes-1, -2, and -3; dicers-1 and -2; loquacious, and aubergine. Expression levels of these transcripts in hemocytes were not affected by bacterial or fungal elicitation of larvae. Targeted depletion of one or more of the above RISC components with double stranded RNAi could disrupt this resistance mechanism rendering pest insects more susceptible to viral infection. Expression of an *H. virescens* transcript orthologous to *C. elegans* SID-1 (Systemic Interference Defective) a transmembrane protein involved in the endocytotic uptake of extracellular double-stranded RNA [45,46] was upregulated by 5.7-fold by bacterial, and 3.1-fold by both fungal. SID-1 activity within insect tissues may be necessary for the success of transgenic plant-based RNAi against feeding insects [47].

### 2.7. Cellular Response Factors

Activation of the immune system by microbial incursion can alter expression of several additional classes of cellular metabolic pathways exemplars of which are presented in Table 4. Glutathione S-transferase, glutathione synthetase, and catalase were upregulated by fungal elicitation; but superoxide dismutase, thioredoxin, thioredoxin peroxidase, and thioredoxin reductase were not significantly upregulated. Cellular oxidative defenses utilized against microbes such as the respiratory burst superoxide generating NADPH-oxidase complex [48] were elevated 4.6-fold by bacterial and 6-fold by fungal elicitation (Table 4). Nitric oxide synthase also was identified, but no significant difference in expression was observed in hemocytes removed from immune elicited larvae. A large number of assemblies orthologous to insect carboxylesterases were identified, some of which are induced by both fungal and bacterial elicitation (Table 4). 

Utilization of the micronutrient iron may shift during infection of *H. virescens* larvae [49,50]. In agreement with this previous work we found that transcript levels for both ferritin and transferrin were elevated by bacterial and fungal elicitation (Table 4). Larval *Bombyx mori* midgut expression of arginine kinase was elevated following BmNPV infection within resistant strains [51], however in this study of *H. virescens* hemocytes expression of arginine kinase was not altered by microbial infection (Table 4). An ortholog of *Bombyx mori* ganglioside-induced differentiation-associated protein was identified [52] but exibited no significant change in transcript level. Three *H. virescens* orthologs of the *Bt*-induced *Spodoptera littoralis* REPAT (Response to Pathogens) were identified [53]. Expression of these orthologs was not altered in hemocytes by bacterial or fungal elicitation of *H. virescens* larvae, though this may have been due to the strains of bacteria used in the present experiments. Enzymes of several classes involved in xenobiotic metabolism were upregulated in hemocytes following infection (Table 4). Of the carboxyl/choline esterase gene family 18 orthologous assemblies were identified along with three acetylcholinesterases. One of the carboxyl/choline esterases identified was upregulated 4.4-fold by bacterial and 5.3-fold by fungal infection (Table 4). Of the 13 UDP-glycosyltransferases detoxification enzyme assemblies none were significantly up- or down-regulated by the treatments used in this study. Over 78 assemblies orthologous to the multidrug resistance proteins of the ATP-binding cassette class were present. Greater than 80 cytochrome P450s were identified, several of which were upregulated either by bacterial or fungal elicitation, or both (Table 4). *H. virescens* larvae defoliate cotton throughout the Americas; thus it was expected that detoxification enzymes which allow feeding on this crop would be identified, though not necessarily in hemocytes. An *H. virescens* ortholog of the *H. armigera* gossypol-induced cytochrome P450 was upregulated 4.5-fold by bacterial elicitation (Table 4).

*H. armigera* larval development was stunted by feeding on transgenic cotton plants expressing double stranded RNAi against this enzyme [54]. *H. virescens* also feeds widely on host plants which deploy glucosinolates as inactive precursors to highly toxic isothiocyanate feeding deterrents. Inactivation of isothiocyanates in insect tissues is achieved by desulphation [55], nitrilases coupled to amidases, or by conjugation to glutathione [56]. Interestingly our hemocyte assembly encoded the detoxification enzyme glucosinolate sulphatase, and the activating enzyme myrosinase, the former of which was upregulated 28-fold by bacterial and 6.8-fold by fungal elicitation of larvae. Nitrilases and amidases also were present though expression was not affected by the treatments employed. Among the other xenobiotic detoxification pathway enzymes were 42 assemblies orthologous to glutathione S-transferases of other Lepidoptera. In other noctuid moths glutathione S-transferases have been demonstrated to detoxify glucosinolates. Two of these GSTs exhibited upregulation following microbial elicitation (Table 4). 

## 3. Experimental Section

### 3.1. Insects, Infection, and RNA Isolation

*Heliothis virescens* eggs were received from the North Carolina State University Dept. of Entomology Insectary (Raleigh, NC, USA). Larvae were reared individually on an artificial wheat germ based diet (BioServ, Frenchtown, NJ, USA) under standard conditions of 14 h:10 h (L:D) photoperiod, 55% RH, 28 °C [23]. To eliminate the possibility of contaminating nucleic acid sequences from injection of live/dead pathogens into experimental insects only purified cell wall components were used to activate the antimicrobial immune response. Early 5^th^ instar larvae were punctured with a tungsten needle dipped into a suspension of 1 µg/mL lipopolysaccharide and 1 µg/mL peptidoglycan (Sigma Chem. Co., St. Louis, MO, USA) in phosphate buffered saline to mimic infection with Gram^−^ and Gram^+^ bacteria [21]. The antifungal response was activated by puncture of early 5^th^ instar larvae with a tungsten needle dipped into a suspension of 1 µg/mL β-glucan, 1 µg/mL curdlan and 1 µg/mL laminarin (Sigma Chem. Co., St. Louis, MO, USA) in PBS. Control larvae were subjected to sterile puncture with a tungsten needle dipped into PBS. At 12 hrs post puncture hemocytes were collected from 30 insects from each treatment according to previously published methods [23]. Larvae were bled through a punctured anterior proleg into ice cold PBS containing a crystal of PTU to prevent melanization. Hemocytes were pelleted by centrifugation at 5000 × *g* for 4 min. and stored at −85 °C. Total RNA was extracted from hemocytes using RNeasy™ kits (Qiagen, Valencia, CA, USA) [23].

### 3.2. Sequence Generation

Hemocyte RNA pools were submitted to University of Missouri Bond Life Sciences Center DNA Core for Illumina GAIIx sequencing (http://www.biotech.rnet.missouri.edu/dnacore/). Libraries were constructed according to the standard Illumina RNA-seq protocol (Part# 1004898 Rev. A, rev Sept 08; http://www.illumina.com) from the pooled PCR products except for the fragmentation step as detailed [23]. 

### 3.3. De Novo Exome Assembly

Illumina reads were first cleaned of low quality bases at the 3’ end, and limited to only those reads longer than 31 bases. The *de novo* assembly was performed using VELVET (v1.0; http://www.ebi.ac.uk/~zerbino/velvet/; 8/29/2011) and OASES (http://www.ebi.ac.uk/~zerbino/oases/; 8/29/2011) with kmers ranging from 21 to 41 in increments of 2. Clusters of contigs with no mismatches were reduced to the longest member using VMATCH (v2.1.4; http://www.vmatch.de/; 8/29/2011). To further reduce redundancy contigs were assembled using TGICL (http://www.compbio.dfci.harvard.edu/tgi/software/; 8/29/2011) to a final count of 103,879. The sequences were annotated by sequence similarity to NR proteins. 

The reads from each sample were aligned to the final set of contigs using SOAP v2 (http://soap.genomics.org.cn/soapaligner.html; 8/29/2011). Contigs were grouped by the NR accession id annotation, and only those Illumina reads were retained that aligned within one and only one such group. Within each group, the expression profile of the sequences was compared one to another and a correlation coefficient generated for each comparison. When two sequences in a group had an r value of 0.8 or more the one with the greater number of hits was retained. The process of measuring the correlation coefficient and winnowing contigs from within a group was continued recursively until left with those sequences whose expression patterns and NR accessions made them unique [57]. In this way all sequences were compared within a group and different expression profiles within each group were retained. The resulting table of differential expression had 22,007 assemblies each with a distinct combination of annotation and expression. Functional categories of contig and singleton sequences resulting from this assembly were annotated using a local installation of BLAST2GO (http://www.blast2go.org; 8/29/2011). All sequences have been deposited (http://www. ncbi.nlm.nih.gov/genomeprj/49697). 

### 3.4. Identification of Differentially Regulated Genes

Larval gene expression was analyzed in a semi-quantitative fashion by normalizing the number of times sequence tags aligning with an mRNA assembly were detected in the hemocytes of infected larvae compared to those of controls. We analyzed genes that were shown to be upregulated or down regulated by a factor of three and that had an e-value < 10^−5^ [23].

## 4. Conclusions

The objective of this effort was to conduct a deep sequence sampling survey utilizing RNA-seq in order to identify as many immune effectors as possible for future studies of immunity with this larval lepidopteran model of entomopathogen infection. Hemocytes are the primary immune responders to microbial infection and this easily collected tissue was expected to express most of the animal’s immune repertoire. Similar deep sampling surveys of other immune responsive tissues with RNA-seq will be needed to round out these initial efforts; however substantial genomic resources have been generated which include the transcripts of major cell surface receptors and signal transduction pathway components. Hemocytes are easily targeted within the hemocoel by injection of gene silencing double stranded RNAi. Sequence resources generated by this study will enable specific, targeted disruption of hemocyte immune system regulatory pathways or other signal transduction pathways *in vivo* [26,32,46,58,59,60,61,62,63]. Importantly, in this study a significant number of differentially regulated transcripts were found which had no identifiable function. These numerous, but unknown transcripts indicate that many aspects of hemocyte function during infection have yet to be discovered. Although restricted to a single tissue, hemocytes, many of these newly identified signal transduction and receptor transcripts, or closely related ones, will also be present in other tissues of *H. virescens*, opening up rich new grounds for studies using this insect model.
